# Characterizing Benzo[a]pyrene Adducts in Transfer RNAs Using Liquid Chromatography Coupled with Tandem Mass Spectrometry (LC-MS/MS)

**DOI:** 10.3390/biomedicines11123270

**Published:** 2023-12-11

**Authors:** Cassandra Herbert, Corinna L. Ohrnberger, Ella Quinlisk, Balasubrahmanyam Addepalli, Patrick A. Limbach

**Affiliations:** Rieveschl Laboratories for Mass Spectrometry, Department of Chemistry, University of Cincinnati, 301 Clifton Court, Cincinnati, OH 45221-0172, USA; herbercr@mail.uc.edu (C.H.);

**Keywords:** post-transcriptional modifications, adductome, B[a]P, BPDE, RNA adducts, modified nucleosides

## Abstract

The activated forms of the environmental pollutant benzo[a]pyrene (B[a]P), such as benzo[a]pyrene diol epoxide (BPDE), are known to cause damage to genomic DNA and proteins. However, the impact of BPDE on ribonucleic acid (RNA) remains unclear. To understand the full spectrum of potential BPDE-RNA adducts formed, we reacted ribonucleoside standards with BPDE and characterized the reaction products using liquid chromatography coupled with tandem mass spectrometry (LC-MS/MS). To understand the potential types of adducts that could form with biological RNAs, eukaryotic transfer RNAs (tRNAs) were also reacted with BPDE. The isolation and analysis of the modified and adducted ribonucleosides using LC-MS/MS revealed several BPDE derivatives of post-transcriptional modifications. The approach outlined in this work enables the identification of RNA adducts from BPDE, which can pave the way for understanding the potential impacts of such adducts on the higher-order structure and function of modified RNAs.

## 1. Introduction

Environment pollutants, such as Polycyclic Aromatic Hydrocarbons (PAHs), have been identified as significant extrinsic factors responsible for the incidence of over 60% of cancer cases [1,2]. This type of pollutant is generated by partial combustion through anthropogenic (e.g., industrial processes and cigarette smoking) and natural (e.g., forest fires and natural petroleum) sources [3]. PAHs are also considered mutagenic and genotoxic [4,5,6]. One PAH, Benzo[a]pyrene (B[a]P), is a typical environmental carcinogen causing lung, liver, and skin cancers [7,8].

**Figure 1:**
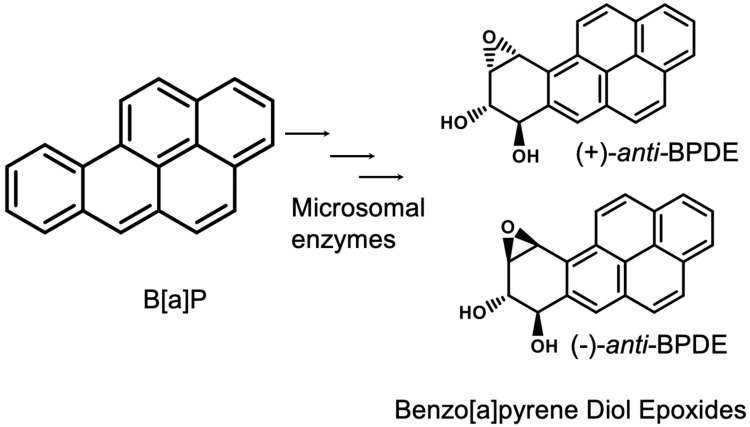
Benzo[a]pyrene and the isomers of its activated form, benzo[a]pyrene diol epoxide (BPDE). The microsomal enzymes that are involved in B[a]P metabolism include mixed-function oxidases and epoxide hydratases.

B[a]P can form diol epoxides through stereospecific oxygenation by microsomal enzymes. Initially, mixed-function oxidases, such as CYP450A1, add oxygen to the 7,8 double bond to form benzo[a]pyrene-7,8-epoxide. This epoxide is hydrated by epoxide hydratase to predominantly yield the trans-7,8-diol or anti-7,8-diol metabolite. This intermediate undergoes another stereoselective oxygenation at the 9,10 double bond via a mixed-function oxidase to form diol epoxide I and diol epoxide II [9,10]. The diols exist as *cis* or *trans* configurational isomers, and each of them can be a (+) or a (−) enantiomer or racemic mixture [11,12] (Figure 1). These isomers of BPDE metabolites are considered mutagenic based on studies with mammalian cells [13], newborn mice [14], and mouse skin [8]. 

BPDE reacts with the native form of DNA at exocyclic amine groups such as the *N*^2^ position of guanine [15] to form (+)-trans- and (−)-cis- or (−)-trans- and (+)cis-dG-*N*^2^–BPDE adducts or the *N*^6^ position of adenine [16] to form dA-*N*^6^–BPDE adducts. DNA damage due to bulky adduction increases the risk of point mutations during error-prone trans-lesional DNA synthesis repair mechanisms at mutational hotspots, which promote carcinogenesis [17,18]. BPDE isomers also form protein adducts at histidine–proline regions and lysine residues [19,20,21]. 

RNA acts as an intermediate in the transfer of genetic information from DNA to proteins, thus acting as a regulatory point during gene expression and cellular responses through interactions with other biomolecules [22,23,24,25]. RNA is also post-transcriptionally modified by cellular enzymes with various chemical groups [26], which in turn modulate the structural and functional properties of RNA [27,28,29,30]. Like DNA, RNA exhibits chemical reactivities towards electrophiles [31]. RNA adducts were documented in greater amounts compared to DNA when cells were exposed to reactive molecules [32,33,34]. More specifically, guanosine adducts of BPDE have been identified using liquid chromatography coupled with tandem mass spectrometry (LC-MS/MS) [35]. 

Cellular quality control mechanisms are expected to degrade adducted RNA. However, any defects or malfunction of such processes can lead to disease conditions [36,37]. It is conceivable that adducts formed on ribosomal RNA (rRNA) (>80% of the total RNA pool in the cell) and transfer RNA (tRNA) (~15% of total RNA pool) could disrupt their structure and function during mRNA translation. In fact, it has been reported that B[a]P exposure led to translational control of gene expression in mouse hepatocytes [38]. Moreover, because the largest amount of RNAs in a cell (i.e., rRNA and tRNA) are known to contain numerous post-transcriptional modifications [26], it is important to understand whether such modified nucleosides are susceptible to B[a]P adduction so that their effects on cellular functions can also be ascertained. Here, we have documented the various ribonucleoside adducts formed by the activated form of B[a]P reacted with tRNAs, which serves as a good model for modified RNAs more generally. These modified ribonucleoside adducts have been identified using LC-MS/MS approaches. We find that most modified ribonucleosides are not susceptible to BPDE adduction.

## 2. Material and Methods

### 2.1. Materials

Isopropanol, ethyl ether anhydrous, acetonitrile (optima LC/MS), and iso-amyl alcohol were purchased from Fisher Scientific (Hampton, NH, USA). Ammonium acetate (LiChropur^®^), dimethyl sulfoxide (microbiology grade), and chloroform (≥99.5%) were purchased from Sigma Aldrich (St. Louis, MO, USA). Tetrahydrofuran (HPLC grade) and ethanol (200 proof) were purchased from Tedia (Fairfield, OH, USA) and Decon Laboratories (King of Prussia, PA, USA), respectively. Tris-HCl was purchased from Research Products Inc. (Theodore, AL, USA). All other chemicals were procured from Thermo Fisher Scientific unless specified.

#### 2.1.1. In Vitro BPDE Exposure of DNA, tRNA and Nucleoside Standards

Baker’s yeast (*Saccharomyces cerevisiae*) tRNA (Sigma Aldrich) and *Escherichia coli* (*E. coli*) DNA (Sigma Aldrich) were exposed to BPDE (Toronto Research Chemicals, Toronto, Ontario, Canada) separately using a procedure similar to the one described previously [39]. Briefly, 50 μg of tRNA or DNA was exposed to 10 μg of BPDE (dissolved in tetrahydrofuran) in ammonium acetate solution (100 mM, 100 μL) at 37 °C overnight in the dark. The tRNA or DNA was extracted with diethyl ether (four times), followed by extraction with iso-amyl alcohol (four times). Subsequently, the tRNA or DNA was precipitated with 2.5 volumes of ethanol overnight at −20 °C. The sample was centrifuged at 12,000× *g* for 15 min, and the resulting pellet was washed with 75% ethanol before solubilization with sterile water. The tRNA or DNA was quantified by measuring the UV absorbance at 260 nm. BPDE reactions with nucleoside standards were performed in the same manner as described above without extraction. 

#### 2.1.2. RNA Hydrolysis to Nucleosides

Defined amounts of treated RNA (5–20 μg) were hydrolyzed to nucleosides as described [40]. Briefly, the denatured RNA (heating at 95 °C for 3 min and snap cooling for 3 min) was incubated with nuclease P1 (0.1 U/μg RNA) (Sigma Aldrich) in 0.01 M ammonium acetate for 2 h. Following neutralization with 1/10 volume of 1 M ammonium bicarbonate, the samples were digested with snake venom phosphodiesterase (1.2 × 10^−4^ U/μg RNA) and bacterial alkaline phosphatase (0.1 U/μg RNA) (Worthington Biochemical corporation, Lakewood, NJ, USA) for 2 h at 37 °C. 

#### 2.1.3. High-Resolution LC-MS/MS-Based Characterization of RNA Adducts from Yeast

The yeast tRNA hydrolysate was dried in speedvac and resuspended in mobile phase A (5.3 mM ammonium acetate, pH 4.5) and subjected to LC-MS/MS. A Vanquish ultra-high performance liquid chromatography (UHPLC, Thermo Scientific, Waltham, MA, USA) system connected to an Orbitrap Fusion Lumos (Thermo Scientific) mass spectrometer was used for data acquisition. Reversed-phase liquid chromatography was performed using a Waters Acquity HSS T3 column (100 Å, 1.8 μm, 1.0 × 100 mm) by employing aqueous ammonium acetate (5.3 mM, pH 4.5) as mobile phase A and 40% acetonitrile as mobile phase B at a flow rate of 100 µL min^−1^. The gradient flow involved 0–2% B at 15.7 min, 3% B at 19.2 min, 5% B at 25.7 min, 25% B at 29.5 min, 50% B at 32.3 min, 75% B at 36.4 min, and 99% B at 39.6 min, followed by switching to 0% B at 46.9 min for equilibration. 

Mass spectra were acquired on the Orbitrap in positive polarity in full scan mode with automatic gain control of 7.5 × 10^4^ and injection time of 100 ms in the mass range of *m/z* 220–900 at 120,000 resolution. The ion funnel radiofrequency was set at 30%, and gas flows were 30 (sheath), 10 (auxiliary), and 0 (sweep) arbitrary units, respectively. The temperature for the ion transfer tube and vaporizer were set to 299 °C and 144 °C, respectively, and the spray voltage was 3.5 kV. Tandem mass spectra were acquired using either low-energy (0–42% ramp) or higher-energy (0–80% ramp) collision-induced dissociation (low-energy CID or HCD) of mass selected ions (isolation width *m/z* 1.6) in an untargeted fashion. Data processing was performed using Xcalibur 3.0 (Thermo Scientific). A mass error of 5 PPM was considered for annotation of the specific ion signal. 

#### 2.1.4. Identification of BPDE Adducts in Exposed Ribonucleoside Standards via SRM LC-MS/MS Analysis

Exposed ribonucleoside standards were subjected to reversed-phase LC-MS/MS analysis using a Vanquish UPLC connected to a TSQ Quantiva Triple Quadrupole mass spectrometer (Thermo Scientific). The ESI spray voltage was set to 3.5 kV, while the sheath, auxiliary, and sweep gas flows were set at 35, 10, and 0 arbitrary units, respectively. The ion transfer tube and vaporizer temperature were set at 200 °C and 260 °C, respectively. Data acquisition was in positive polarity using the selected reaction monitoring (SRM) mode, where the resolution for Q1 and Q3 were set at 0.7 with a dwell time of 300 ms. CID fragmentation was performed at 1.5 mTorr nitrogen. Adduct detection was performed by monitoring the mass transitions involving nucleoside-adduct molecular ions and defined product ions identified in the high-resolution LC-MS/MS analyses (Supplementary Table S1). LC-MS data processing was performed with Xcalibur 3.0 (Thermo Scientific).

## 3. Results and Discussion

### 3.1. LC-MS Based Characterization of Ribonucleoside–BPDE Adducts

Stereoselectivity and the distribution of BPDE adducts are modulated by the primary nucleotide sequence, thereby introducing bias towards certain bases (such as tandem guanine bases adjacent to adenine) [41] due to stereoelectronic effects of nucleobases and the stereochemistry of BPDE isomers [14]. Therefore, a variety of BPDE adducts might be anticipated upon reaction between intact RNAs, containing a variety of chemical groups and BPDE. Transfer RNA, which is heavily post-transcriptionally modified, serves as a good model to investigate the types of adducts that can be formed between BPDE and modified RNAs.

**Figure 2:**
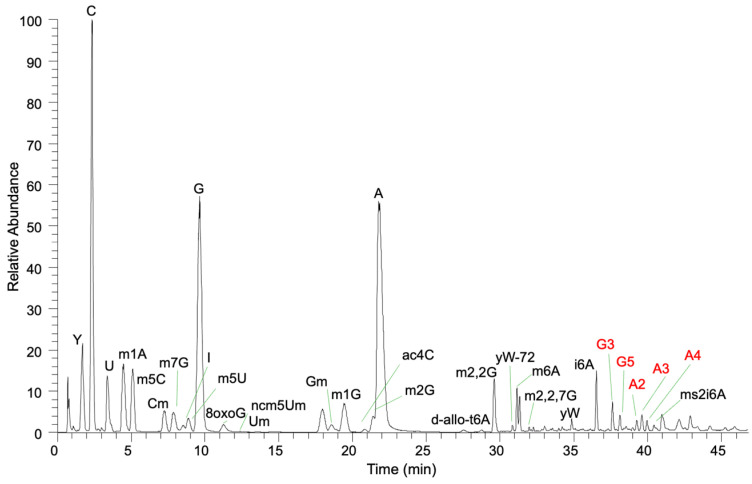
Total ion chromatogram of yeast tRNA exposed to BPDE. Modified nucleosides and BPDE adducts (guanosine and adenosine) are labeled.

To document the types of RNA-B[a]P adducts formed on post-transcriptionally modified RNA, yeast total tRNA was directly exposed to BPDE, and the resulting RNA adducts were characterized using LC-MS/MS in an untargeted fashion (Figure 2). Putative BPDE adducts were determined by searching for the anticipated (ribonucleoside + adduct) mass value (Supplementary Table S2). Extracted ion chromatograms for each *m/z* value were then generated. The presence of BPDE-specific product ions was considered to be a diagnostic signal for the adduct assignment. For each ribonucleoside present in yeast that was believed to contain a BPDE adduct, those adducts were confirmed by conducting reactions with nucleoside standards that were then analyzed in a targeted MS/MS approach. Adducts for the canonical ribonucleosides are presented, followed by those detected for modified ribonucleosides.

**Figure 3:**
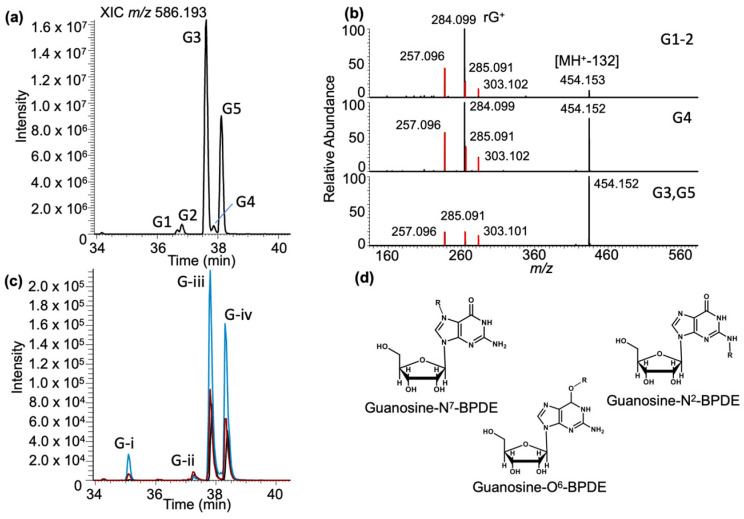
Characterization of the guanosine–BPDE adduct based on LC-MS analysis. (**a**) XIC of rG-BPDE at *m/z* 586.193. Chromatographic peaks at retention times of 36.67, 36.90, 37.60, 37.86, and 38.10 min are labeled G1–5. (**b**) Representative MS/MS spectrum of CID fragmentation of *m/z* 586.193 from defined XIC peaks (labeled on the right). Fragments include the loss of the ribose group [M-132] *m/z* 454.152, guanosine nucleoside (Guan+) and fragments of the BPDE (*m/z* 257.096 and 303.102). (**c**) Guanosine exposed to BPDE at varying pHs: pHs 4 (black trace), 6 (red trace), and 9 (blue trace). Four peaks eluted at the retention times of 35.10, 37.29, 37.82, and 38.31 min labeled (G-)i–iv. (**d**) The proposed structures of rG–BPDE adducts, which shows the attachment of adduct (R) at N^7^, N^2^ and O^6^ positions of guanosine.

***Guanosine–BPDE adducts***: Analysis of the LC-MS data for guanosine–BPDE adduct molecular ion (MH^+^, *m/z* 586.193) revealed five peaks (labeled as G1–G5) in the extracted ion chromatogram (XIC) eluting between 36.6 and 38.1 min (Figure 3a). The MS/MS spectra of these peaks revealed the presence of BPDE-specific product ions: *m/z* 257.096, 285.091, and 303.102 (Supplementary Figure S1) as described before [35]. However, the most abundant product ions in the MS/MS spectra differed for these adducts (Figure 3b). The most abundant product ion for G1 and G2 was *m/z* 284.099, which corresponds to the guanosine nucleoside and suggests the loss of the BPDE adduct (−302 Da). In contrast, the most abundant product for G3 and G5 was *m/z* 454.151, which corresponds to the loss of ribose sugar (−132 Da). Interestingly, both the *m/z* 454.151 and *m/z* 284.099 product ions were observed at high abundance for G4. A compilation of all MS/MS product ions and their assignment as BPDE fragments, loss of BPDE adduct, and loss of ribose are found in Supplementary Table S3. 

To confirm that these adducts arise from guanosine and not some other components in the tRNA sample, BPDE was reacted with a standard solution of guanosine at three different pH values (4, 6, and 9). Selected reaction monitoring (SRM) was used to recapitulate the untargeted results from yeast (Supplementary Table S1). As seen in Figure 3c, the general adduct profile for the nucleoside standard is similar to that found in yeast tRNAs. Two major adducts, corresponding to G3 and G5, are detected along with several lower-abundance adducts. There was a notable difference in adduct abundance at pH 9, which likely is accounted for by the reactivity of different adduction sites on guanosine. Overall, guanosine had the highest ion abundance detected for the BPDE-containing adducts (Supplementary Figure S2). Differences in retention time, adduct abundance, and MS/MS product ion spectra suggest that these five peaks are different positional isomers (e.g., adduction at *N*^7^, *N*^2^ or O^6^, Figure 3d) or stereoisomers [11,12,42]. 

BPDE adducts of guanosine have previously been resolved and detected using liquid or gas chromatography in combination with mass spectrometry [19,43,44]. Thus, it is not surprising to detect multiple BPDE adduct peaks. While the structural elucidation of each individual ribonucleoside + BPDE adduct is beyond the scope of this work, the differences in dissociation patterns (loss of ribose sugar (MH^+^-132) vs. the entire adduct (MH^+^-BPDE)) could indicate inherent bond dissociation preferences for N^2^ and O^6^–BPDE adducts, respectively. 

**Figure 4:**
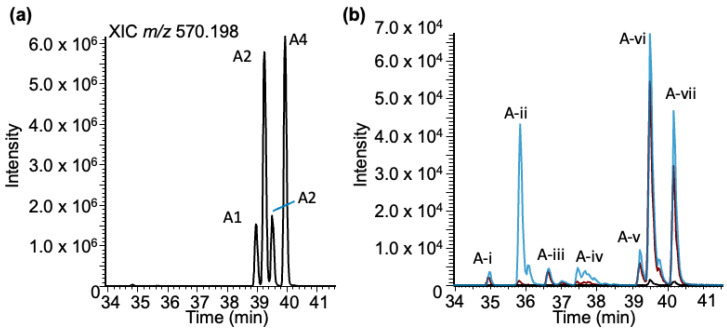
Characterization of the BPDE adduct formed with adenosine (rA-BPDE) based on LC-MS. (**a**) XIC of rA-BPDE at *m/z* 570.198. (**b**) Adenosine exposed to BPDE at varying pHs: pHs 4 (black trace), 6 (red trace), and 9 (blue trace). Seven peaks are labeled at the retention times: 34.94, 35.86, 36.63, 37.50, 39.24, 39.52, and 40.19, mins labeled (A-)i–vii.

***Adenosine–BPDE adducts***: Analysis of the LC-MS data for adenosine–BPDE adduct molecular ion (*m/z* 570.198) revealed four peaks (labeled as A1–A4) in the extracted ion chromatogram (XIC) eluting between 38 and 40 min (Figure 4a). However, unlike the case for guanosine, the MS/MS spectra of all these peaks exhibited the same product ion spectra with product ions representing BPDE (*m/z* 257.096, 285.091 and 303.102), adenosine (*m/z* 268.103), and loss of ribose sugar (MH^+^-132, *m/z* 438.155) (Supplementary Figure S3). As before, to confirm that these adducts arise from adenosine, a standard solution was reacted with BPDE at three pH values and analyzed using a targeted SRM approach. 

For the BPDE-exposed adenosine standard, more total peaks (six) were detected than were found from the yeast tRNA sample (Figure 4b). One adduct (A-ii), eluting at 35.9 min, was only detected with any significance at pH 9. This peak has similar characteristics (decrease in retention time and increase in ion abundance correlated with an increase in pH) to the early eluting peak identified in the G-BPDE standard sample. These results may indicate that early eluting peaks that are sensitive to basic reaction conditions may represent an adduct occurring at the N7 position of each purine. Adduction at that position is expected to result in a positively charged nucleoside, which would reduce retention time under these reversed-phase HPLC conditions. 

**Figure 5:**
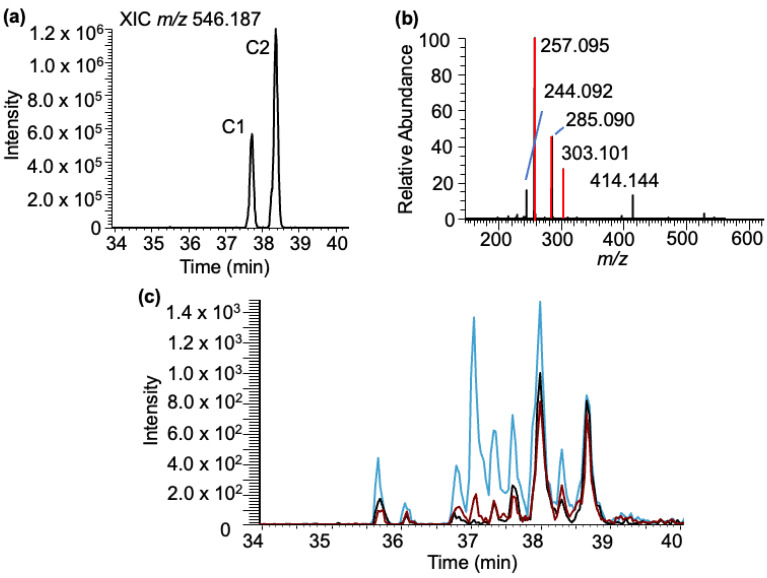
Characterization of the cytidine–BPDE adduct based on LC-MS. (**a**) XIC of rC-BPDE at *m/z* 546.187. (**b**) MS/MS spectrum of the molecular ion *m/z* 546.187. Fragments include the loss of the ribose [M-132] *m/z* 414.144 and cytidine ion *m/z* 244.092, and the BPDE-specific fragment ions (*m/z* 257.095, 285.090, 303.101) are labeled. (**c**) Cytidine exposed to BPDE at varying pHs: pHs 4 (black trace), 6 (red trace), and 9 (blue trace).

***Cytidine–BPDE adducts***: Analysis of the LC-MS data for cytidine–BPDE adduct molecular ion (*m/z* 546.187) revealed two peaks (labeled as C1–C2) in the extracted ion chromatogram (XIC) eluting between 37 and 39 min (Figure 5a). Tandem mass spectra of the molecular ion revealed the presence of minor product ions corresponding to cytidine (*m/z* 244.092) and the loss of the ribose sugar (*m/z* 414.144). The other major product ions from both adducts arise from BPDE-specific products (Figure 5b). 

Again, to confirm that these adducts arise from cytidine, a standard solution was reacted with BPDE at three pH values and analyzed using a targeted SRM approach. Unlike the results found with the purine canonicals, significantly different results were found with the cytidine standard (Figure 5c). Up to nine discrete cytidine–BPDE adduct ions (*m/z* 546.187) were detected in the elution window between 35 and 39 min. As with guanosine and adenosine, reactions conducted at pH 9 tended to result in increasing ion abundance for many of the cytidine–BPDE adducts, but many of the adducts were observable at pH 4 and were detected with relatively higher abundance compared to pH 6 (Supplementary Figure S2). Of general note, cytidine–BPDE adducts from these confirmatory experiments were generally of lower abundance than those with the purine standards, which is not unexpected given the known reactivity of purines to BPDE adduction. Given the fewer reactive sites in the pyrimidine ring, some of these adducts may be occurring on the ribose (i.e., 3′- or 5′-hydroxyl) that are unavailable for adduction in the polynucleotide chain. Unlike G-BPDE, the bond dissociation pattern indicated by MS/MS does not suggest a possibility of the adduct occurring at a position specific to the N^4^ or O^2^ position of cytosine, as the relative abundance of fragment ions that correspond to ribose loss or BPDE loss is more or less equal. 

***Uridine–BPDE adducts***: No discrete molecular ions corresponding to uridine–BPDE adducts (*m/z* 547.170) were detected beyond isotopic contributions from cytidine–BPDE adducts. 

***Deoxyribonucleoside–BPDE adducts***: To determine how the ribonucleoside–BPDE adducts compare to deoxyribonucleoside adducts, the same treatment conditions were applied to *E. coli* DNA, and deoxyribonucleoside adducts were analyzed using LC-MS/MS (Supplementary Figure S4). Adduct profiles for dC (Supplementary Figure S4A), dA (Supplementary Figure S4C), and dG (Supplementary Figure S4E) are generally similar to their ribonucleoside counterparts. Additionally, comparing the relative levels of all nucleoside-specific BPDE adducts showed similar trends for both deoxyribonucleosides and ribonucleosides (Supplementary Figure S5), with dG/G being the most reactive and dC/C being the least reactive. Further, while not rigorously quantitative, it appears the ribonucleoside purines are more reactive to BPDE than their deoxyribonucleoside counterparts.

### 3.2. LC-MS-Based Characterization of Modified Ribonucleoside–BPDE Adducts

While ribonucleoside–BPDE adducts were found for guanosine, adenosine, and cytidine, a different pattern emerged with modified ribonucleosides. An examination of the predicted *m/z* value for each modified ribonucleoside + BPDE adduct (Supplementary Table S2) revealed that only two major classes of modified ribonucleosides from yeast tRNA, inosine and methylated guanosines, were found to generate detectable levels of BPDE adducts. Interestingly, no BPDE adducts were detected for the double-methylated guanosine, m^2,2^G. Furthermore, no adducts were found for modified pyrimidines or the complex modified nucleosides known in yeast, such as wybutosine and queuosine. 

**Figure 6:**
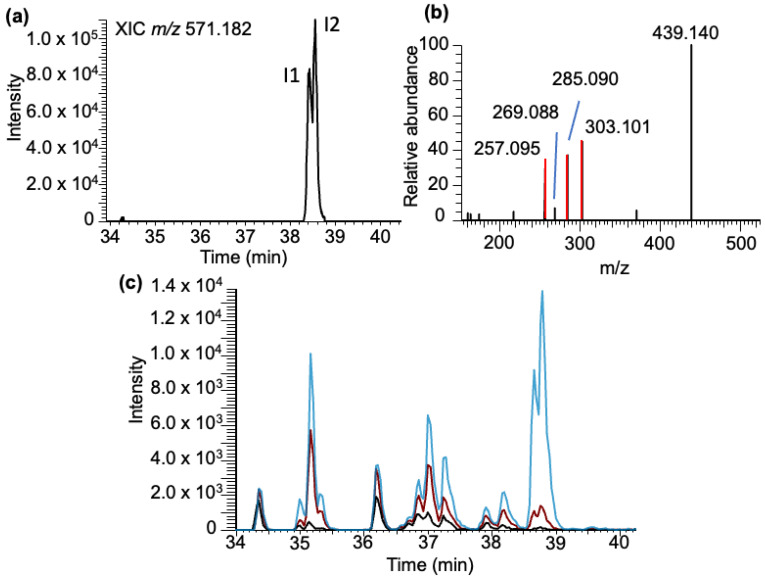
Characterization of the BPDE adduct formed with inosine (I-BPDE). (**a**) XIC of I-BPDE at *m/z* 571.182. (**b**) MS/MS spectrum of molecular ion following CID of molecular ion *m/z* 571.182 is shown. Fragments include the loss of the ribose group [M-132] *m/z* 439.140, MH^+^-BPDE corresponding to inosine with *m/z* 269.089, and the BPDE-specific fragment ions (*m/z* 257.095, 285.090, and 303.101) are labeled. (**c**) Inosine nucleoside standard exposed to BPDE at varying pHs: pH 4 (black trace), 6 (red trace), and 9 (blue trace).

***Inosine–BPDE adducts***: Analysis of the LC-MS data for inosine–BPDE adduct molecular ion (*m/z* 571.183) revealed two closely eluting peaks (labeled as I1 and I2) in the extracted ion chromatogram (XIC) eluting at 38.43 and 38.56 min (Figure 6a). Their elution time is different from that of adenosine–BPDE adducts, indicating they are unique inosine–BPDE adducts, and the results observed in the XIC do not arise from the ^13^C isotope of adenosine–BPDE adducts. Tandem mass spectra of these peaks revealed the major product ion to be associated with the loss of ribose (*m/z* 439.140), with a very minor product ion corresponding to inosine (*m/z* 269.088). The other significant product ions (*m/z* 303.101, 285.090, and 257.095) arise from BPDE-specific fragment ions (Figure 6b). 

As with the canonicals, a standard solution of inosine was reacted with BPDE, and the reaction mixture was analyzed in targeted MS/MS fashion via SRM. Unlike the guanosine or adenosine reactions, a significant number of BPDE adducts were identified across a broad elution window (34–39 min, Figure 6c). Reactions conducted at pH 6 and 9 resulted in significant increases in the inosine–BPDE adduct abundance across all adducts in comparison to those performed at pH 4. Additionally, the abundance of adducts detected suggests that inosine has a relatively higher amount of adduct formed compared to all detected nucleoside BPDE adducts apart from G-BPDE (Supplementary Figure S2). This result is potentially due to the large variety of potential adducts formed with the nucleoside standard. Although the specific identity of each inosine–BPDE adduct has not been determined, the limited adduction found in the yeast tRNA data does suggest that the reactivity of inosine differs significantly at the nucleoside level as compared to its integration into a polynucleotide chain.

**Figure 7:**
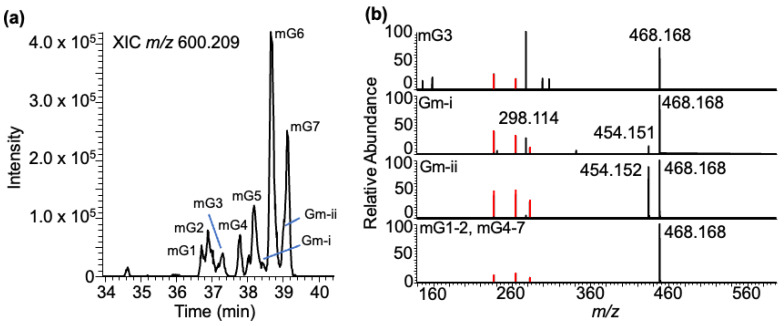
Characterization of the BPDE adduct formed with the methyl-guanosine (mG). (**a**) XIC of mG-BPDE at *m/z* 600.209. Peaks labeled mG1-7 are retained at 36.72, 36.90, 36.99, 37.20, 38.20, 38.67, and 39.12 min. (**b**) Tandem mass spectra of defined XIC peaks following CID are shown. Methylated guanosine ion with *m/z* 298.114, loss of methylated ribose [MH^+^-146] with *m/z* 454.148, loss of ribose [M-132] with *m/z* 468.165, and the adduct-specific fragment ions are labeled. Peaks Gm-i and Gm-ii indicate the XIC peaks containing the Gm–BPDE adducts based on the loss of methylated ribose from molecular ion in the MS/MS analysis.

***Methylated guanosine–BPDE adducts***: Yeast tRNA contains several different methylated guanosines, where the position of methylation can vary on the nucleobase or arise from ribose methylation. The adducts of these positional isomers (m^2^G, m^1^G, and m^7^G) or ribose methylation (Gm) are expected to generate molecular ions with identical *m/z* values (*m/z* 600.209). Analysis of the LC-MS data at this *m/z* value yielded seven peaks (mG1-7) eluting between 36 and 40 min (Figure 7a). Apart from the BPDE-specific fragment ions, *m/z* 303.101, 285.090, and 257.096, the tandem mass spectra contained product ions such as *m/z* 468.166 (loss of ribose from mG + BPDE adduct), *m/z* 454.148 (Gm + BPDE minus loss of 2′-O-methylribose (146.058)), and *m/z* 298.115 (corresponding to protonated mG resulting from the loss of BPDE). While the loss of ribose (−132 Da) was noticed for almost all XIC peaks, a loss of 146 Da (methylated ribose) was noticed in the regions labeled as Gm-I and Gm-ii, indicating that these two regions contained Gm + BPDE (versus mG + BPDE) adducts (Figure 7b).

**Figure 8:**
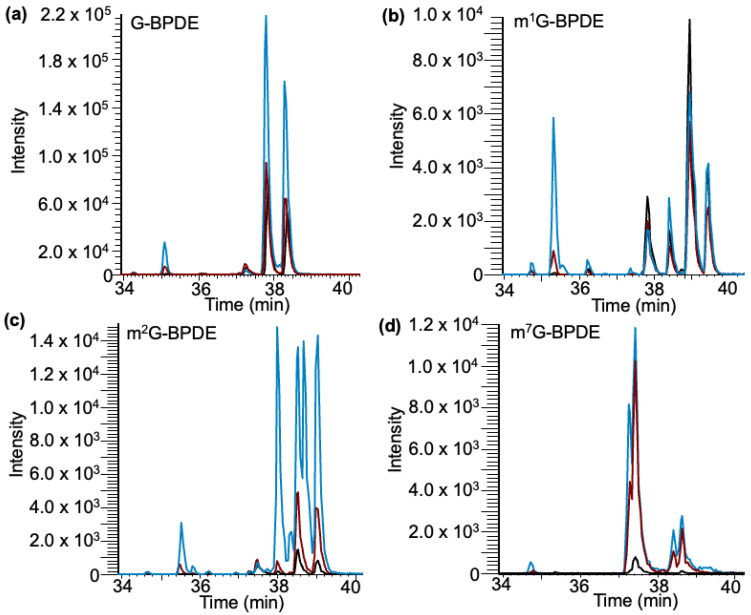
Detection of canonical and methylated G–BPDE adducts in nucleoside standards after exposure to BPDE through selected reaction monitoring. Exposures performed at varying pHs of 4 (black trace), 6 (red trace), and 9 (blue trace). (**a**) Detection of G–BPDE adducts containing 5 peaks. (**b**) Detection of m1G-BPDE containing 5 peaks. (**c**) Detection of m2G–BPDE adducts containing 6 peaks. (**d**) Detection of m7G–BPDE adducts containing 4 peaks.

Based on our analysis of the yeast data, three nucleoside standards were evaluated for mG–BPDE adduct formation: m^1^G, m^2^G, and m^7^G. Through LCMS/MS analysis, several peaks were identified, which differed among the various nucleoside standards (Figure 8). The time frame of elution of peaks coincides with previous data collected in the in vitro exposure of BPDE to yeast tRNA (36.5–39.5 min). As occurred with all standards, adduct peaks were most abundant at pH 9 and generally of lowest abundance at pH 4 (Supplementary Figure S2). 

Interestingly, G, m^1^G, and m^2^G had a peak that eluted around 35.5 min, which is most abundant at pH 9, whereas m^7^G did not generate a peak around that retention time. As previously stated, early elution may be an indicator of a charge deposited on the N^7^ due to alkylation at that position, which would explain why both m^1^G and m^2^G generate such an adduct while m^7^G does not. The calculated pKa for the N^7^ position on all Gs exposed is 2.2–3.3, which would give the site a higher potential for adduction [45]. 

**Figure 9:**
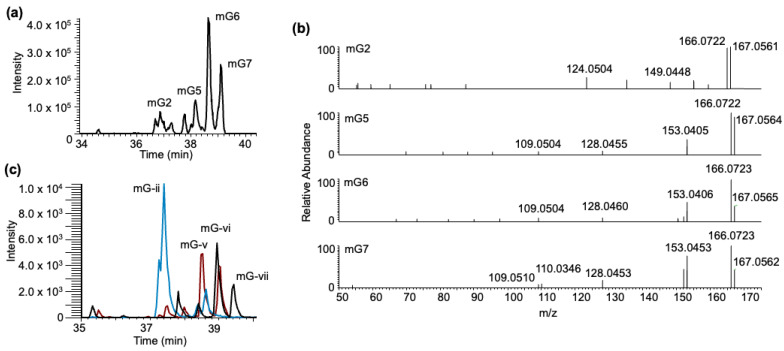
HCD fingerprinting of mG–BPDE adducts. (**a**) XIC chromatogram of mG–BPDE adducts after tRNA exposure, highlighting previously annotated peaks, mG2, mG5-7 at *m/z* 600.209. (**b**) HCD fragmentation profiles of annotated peaks. (**c**) Overlay of mG–BPDE adducts detected via SRM after m^1^G (black trace), m^2^G (red trace), and m^7^G (blue trace) ribonucleoside standard exposure to BPDE at pH 6, highlighting 4 peaks, (mG-)ii, v–vii.

To potentially differentiate the isomers of guanine methylations in high-resolution full scan data, we adopted higher-energy collision dissociation (HCD) to identify the presence of methylated nitrogenous base isomer-specific fragment ions in the tandem spectra [40] as shown in Figure 9. Such analysis indicated a tentative assignment of XIC peak mG2 to m^7^G-BPDE (due to the presence of *m/z* 124.050). Peaks (mG) 5–7 contained a similar pattern of fragment ions that are expected for m^1^G-BPDE (due to the presence of *m/z* 166.072, 153.042, 109.051), with peak mG7 containing the additional identifying fragment (*m/z* 110.0346) (Figure 9b). The overlayed chromatograms of the SRM analysis of m^1^G, m^2^G, and m^7^G ribonucleoside standards after BPDE exposure produced a similar profile to the one after tRNA exposure (Figure 9c). Peak mG- ii, containing mainly m^7^G-BPDE, elutes at a similar position as mG2, in which m^7^G was identified through HCD analysis. Peaks mG5-7 were assigned to m^1^G; however, m^2^G has a similar fragmentation profile. There is only one differentiating peak generally found at 57.0453. However, in the profile, this peak is found in relatively low abundance [40].

These results from a mixture of yeast total tRNAs shows that few modified ribonucleosides have a strong propensity to react and form BPDE adducts. Those modified ribonucleosides that do form adducts (inosine and methylated guanosines) most likely occur based on the inherent chemical reactivity of purines with electrophiles. Inosine, which does form adducts with BPDE, is found in the anticodon. However, there were no complex modified nucleosides from the anticodon of yeast, such as wybutosine or *N*^6^-threonylcarbamoyladenosine (t^6^A), that were found to form adducts. Moreover, the various methylated guanosines (m^1^G, m^2^G, and Gm) are found throughout the sequence of yeast tRNAs. While this work did not focus on determining the specific structures of these BPDE adducts, it seems likely that all possible methylguanosines found in yeast can form adducts.

Further, these data do not suggest that BPDE adduction is a function of the overall level of modified nucleosides in yeast tRNAs, as dihydrouridine and pseudouridine are two of the most abundant modifications (being present in the D-loop and TYC-loop, respectively) as they are present in all yeast tRNAs. Despite their relatively higher concentration within the overall mixture, these modifications arise from the uridine canonical, which was found to be unreactive to BPDE in this work.

These data do not rule out the possibility that the tertiary structure of tRNAs has an effect on adduction via BPDE. BPDE is a bulky adduct that must access appropriate exocylic sites on purines (primarily guanosine) for reaction. The tertiary structure of tRNAs, including intermolecular base-pairing, likely limits the number of nucleoside residues for reaction. While beyond the scope of this work, it would be of interest to perform RNA modification mapping [46] to identify whether specific regions of tRNAs are more prone to BPDE adduction than others in a manner similar to that found for oxidative damage of RNAs [47].

## 4. Conclusions

We have documented the formation of a variety of ribonucleoside–BPDE adducts and their potential diastereomers in yeast tRNA and ribonucleoside standards exposed to BPDE. Our findings suggest that BPDE adduction in modified RNAs, similar to what is found for other biomolecules, is primarily driven by the chemical nature of the substrate (e.g., purines are more reactive than pyrimidines) with the overall amount of each canonical and modified nucleoside playing a secondary role (hence the presence of cytidine-BPDE adducts). Overall, while RNA may have a greater propensity to react with electrophiles such as BPDE, there is no evidence that modified nucleosides, at least in tRNAs, contribute significantly nor serve as any “hotspots” for adduction. Future work focused on identifying specifical locations for tRNA adduction can advance our understanding of their effects on RNA structure and function.

## Data Availability

LC-MS/MS data in non-proprietary format are available upon request of the authors.

## Supplementary Materials

The following supporting information can be downloaded at: https://www.mdpi.com/article/10.3390/biomedicines11123270/s1, Figure S1: BPDE fragments of guanosine + BPDE adducts; Figure S2: Relative quantification of potential nucleoside adducts at pH 4 (white), 6 (red) and 9 (black); Figure S3: Adenosine- BPDE adducts; Figure S4: DNA-BPDE adduct characterization via LC-MS/MS; Figure S5: Relative quantification of deoxyribonucleoside and ribonucleoside BPDE adducts; Table S1: Selected Reaction Monitoring (SRM) transitions monitored for each ribonucleoside + BPDE standard; Table S2: Calculated and observed ribonucleoside + BPDE adduct molecular ions; Table S3: Classification of the detected CID product ions from ribonucleoside + BPDE adducts.
