# Supplementary material for: Characterizing Benzo[a]pyrene Adducts in Transfer RNAs Using Liquid Chromatography Coupled with Tandem Mass Spectrometry (LC-MS/MS)
Source: Biomedicines. 2023 Dec 11;11(12):3270. doi: 10.3390/biomedicines11123270 (PMC10741534; doi:10.3390/biomedicines11123270)
Supplement: Supplementary file 1 [file biomedicines-11-03270-s001.zip › biomedicines-2738206-supplementary.pdf]

SUPPLEMENTAL INFORMATION FOR

Article

Characterizing Benzo[a]pyrene Adducts in Transfer RNAs using Liquid Chromatography Coupled with Tandem Mass Spectrometry (LC-MS/MS)

Cassandra Herbert, Corinna L. Ohrnberger, Ella Quinlisk, Balasubrahmanyam Addepalli and Patrick A. Limbach *

Rieveschl Laboratories for Mass Spectrometry, Department of Chemistry, University of Cincinnati, 301 Clifton Court, Cincinnati, OH 45221-0172, USA; herbercr@mail.uc.edu (C.H.);
* Correspondence: pat.limbach@uc.edu; Tel.: +1-(513)-558-0026; Fax: +1-(513)-556-9239

Contents

Supplemental Figures S1-S5

Supplemental Tables S1-S3

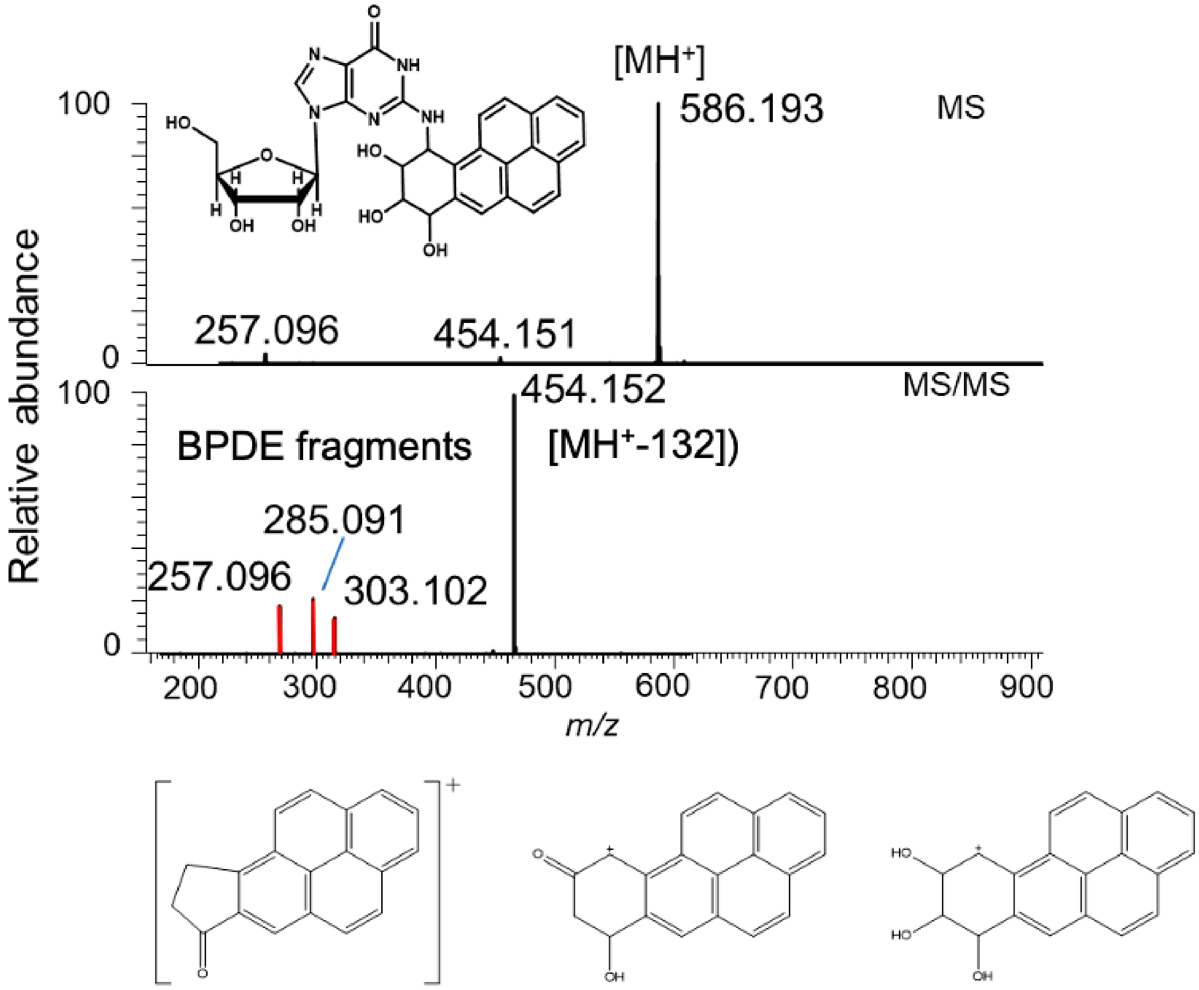

m/z 257.0960 *m/z* 285.0911 *m/z* 303.1015

Supplemental Figure S1. BPDE fragments of guanosine+BPDE adduct. Mass-spectrum (MS) of the XIC for *m/z* 586.192 (guanosine+BPDE) is shown in the top panel. MS/MS spectrum of the molecular ion *m/z* 586.192 following collision-induced dissociation (CID) is shown in the bottom panel. BPDE-specific fragment ions (*m/z* 257.096, 285.091 and 303.101), ribose (132 Da) loss (*m/z* 454.152) from molecular ion are depicted. Putative structures of BPDE-specific fragment ions are also shown.

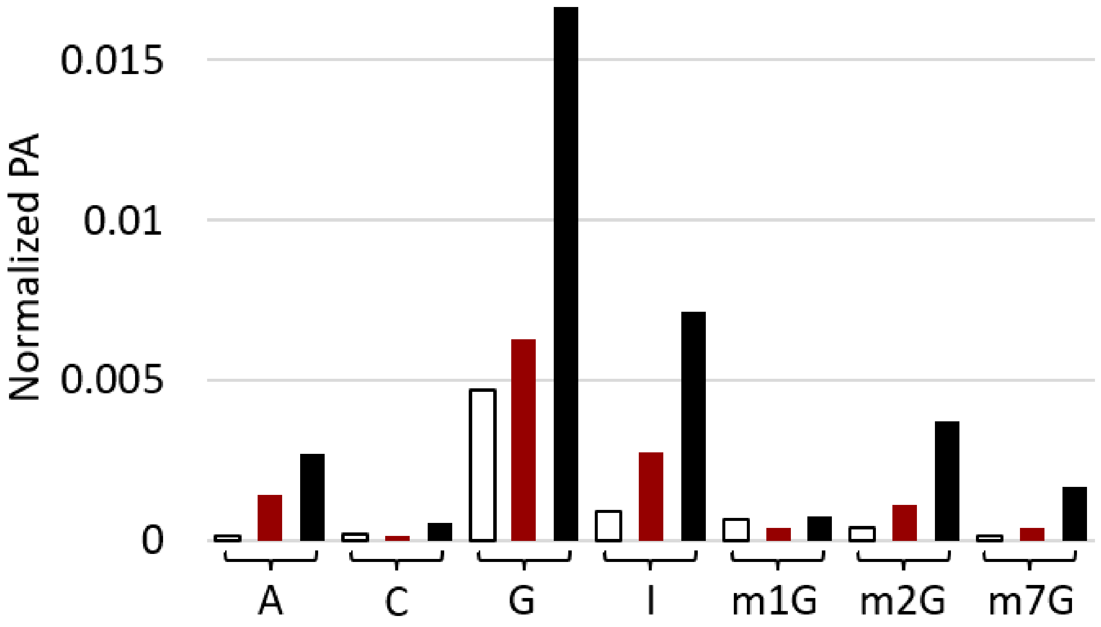

Supplemental Figure S2. Relative quantification of potential nucleoside adducts at pH 4 (white), 6 (red) and 9 (black). Peak areas of the summation of all adduct peaks were normalized to the total abundance of ions detected in both the adducts and the canonical nucleoside.

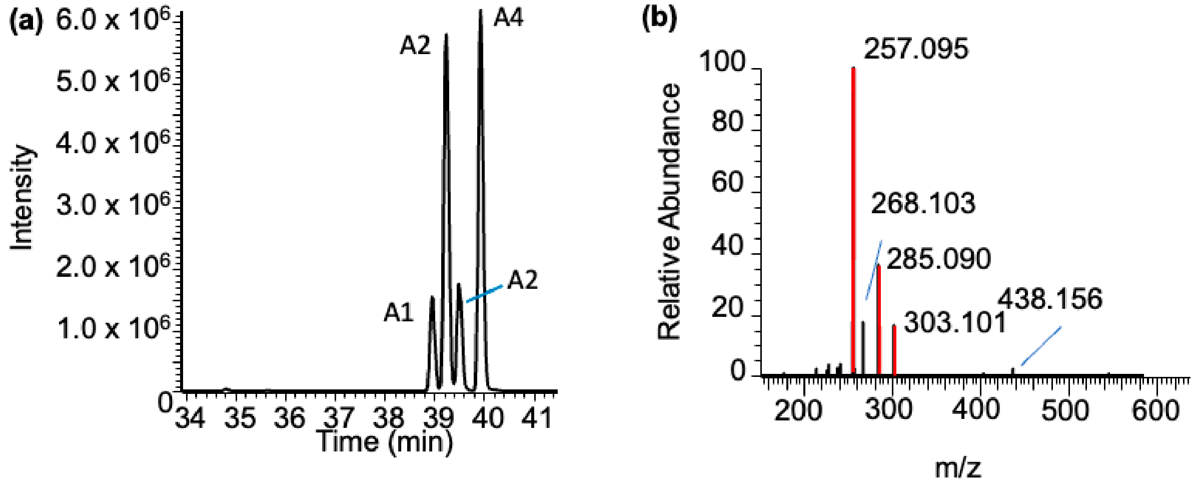

Supplemental Figure S3. Adenosine- BPDE adducts. (a) Extracted ion chromatogram for *m/z* 570.198. (b) MS/MS spectrum of the molecular ion *m/z* 570.198 following collision-induced dissociation (CID) is shown. BPDE-specific fragment ions (*m/z* 257.096, 285.090 and 303.101), ribose (132 Da) loss (*m/z* 438.156) from molecular ion are depicted.

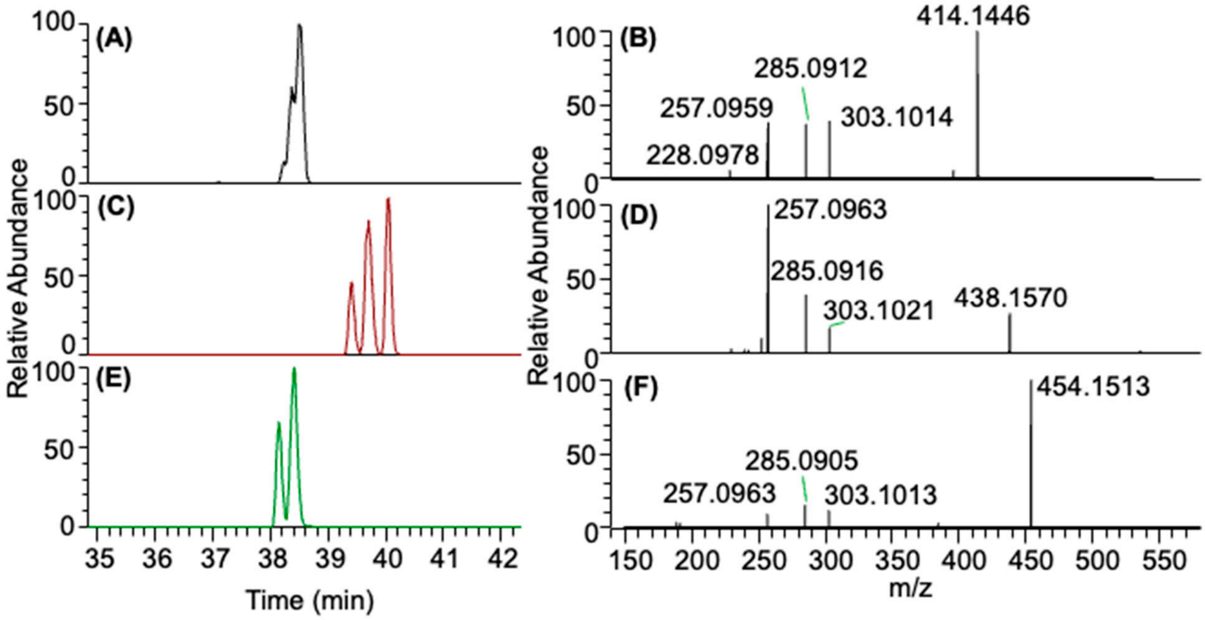

Supplemental Figure S4. DNA-BPDE adduct characterization via LC-MS/MS. (**A**) XIC of dC-BPDE, *m/z* 530.1921 and (**B**) MS/MS profile. (**C**) XIC of dA-BPDE, *m/z* 554.2033 and (**D**) MS/MS profile. (**E**) XIC of dG-BPDE, *m/z* 570.1980 and (**F**) MS/MS profile.

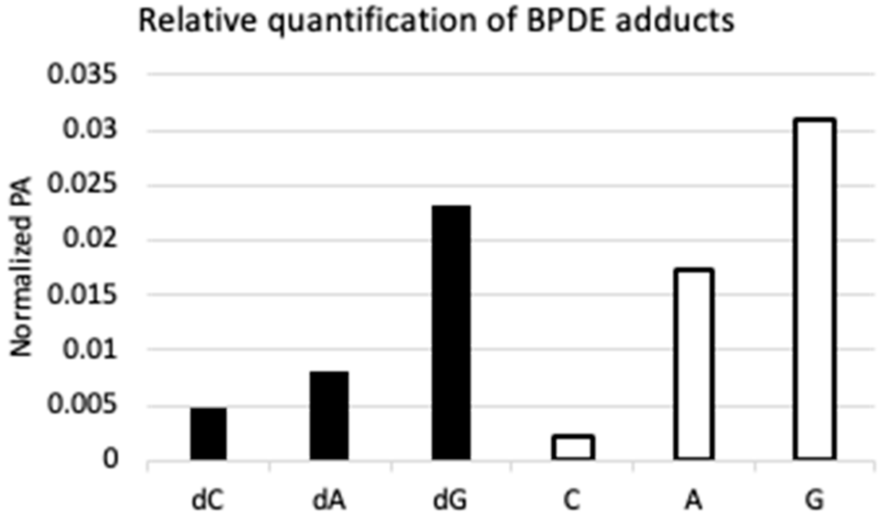

Supplemental Figure S5. Relative quantification of deoxyribonucleoside and ribonucleoside BPDE adducts. Peak areas of the summation of all adduct peaks were normalized to the total abundance of ions detected in both the adducts and the canonical (deoxy)ribonucleoside.

Supplemental Table S1: Selected Reaction Monitoring (SRM) transitions monitored for each ribonucleoside+BPDE standard.

Compound	Retention Time (min)	RT Window (min)	Polarity	Precursor (m/z)	Product (m/z)
BPDE	36	24	Positive	303	257
BPDE	36	24	Positive	303	285
C_BPDE	36	24	Positive	546	244
C_BPDE	36	24	Positive	546	257
C_BPDE	36	24	Positive	546	285
C_BPDE	36	24	Positive	546	303
C_BPDE	36	24	Positive	546	414
A_BPDE	36	24	Positive	570	257
A_BPDE	36	24	Positive	570	268
A_BPDE	36	24	Positive	570	285
A_BPDE	36	24	Positive	570	303
A_BPDE	36	24	Positive	570	438
I_BPDE	36	24	Positive	571	257
I_BPDE	36	24	Positive	571	269
I_BPDE	36	24	Positive	571	285
I_BPDE	36	24	Positive	571	303
I_BPDE	36	24	Positive	571	439
G_BPDE	36	24	Positive	586	257
G_BPDE	36	24	Positive	586	284
G_BPDE	36	24	Positive	586	285
G_BPDE	36	24	Positive	586	303
G_BPDE	36	24	Positive	586	454
mxG_BPDE	36	24	Positive	600	275
mxG_BPDE	36	24	Positive	600	285
mxG_BPDE	36	24	Positive	600	298
mxG_BPDE	36	24	Positive	600	303
mxG_BPDE	36	24	Positive	600	468

Supplemental Table S2: Calculated and observed ribonucleoside+BPDE adduct molecular ions.

Ribonucleoside	MH+ (Theoretical)	MH+ (Observed)	Retention time(s) (min)
C	546.1859	546.187	37.74/38.39
A	570.1971	570.1982	39.98/39.25/39.52/39.94
I	571.1811	571.1823	38.42/38.55
G	586.192	586.1929	36.67/37.60/37.86/38.10
m^2G	600.2077	600.209	39.12
Gm	600.2077	600.209	38.67/39.12
m^1G	600.2077	600.209	36.72/36.99/37.30/37.78/38.20
m^7G	600.2077	600.209	36.9
U	547.1699		
Ψ	547.1699		
D	549.1855		
m^5C	560.2015		
m^3C	560.2015		
Cm	560.2015		
Um/m^3U	561.1855		
m^5U	561.1855		
Am	584.2127		
m^6A	584.2127		
m^1A	584.2127		
m^1I	585.1968		
ac^4C	588.1964		
$m^{6,6}A$	598.2284		
8oxoG	602.1869		
ncm^5U	604.1913		
$m^{2,2}G$	614.2233		
ncm^5Um	618.207		

mcm^5U	619.191
$m^{2,2,7}G$	628.239
mcm^5s^2U	635.1682
i^6A	638.2597
ms^2i^6A	684.2474
L-t^6A	715.2346
d-allo-t^6A	715.2346
yW-72	739.271
yW	811.2921

Supplemental Table S3. Classification of the detected CID product ions from ribonucleoside+BPDE adducts.

Ribo-nucleoside	MH+ (Theoretical)	MH+ (observe)	Retention time (min)	CID product ions		
				BPDE-fragment ions	w/out BPDE adduct	w/out ribose
C	546.1859	546.187	37.74/38.39	257.0953/285.0903/303.1009	244.092	414.1445
A	570.1971	570.1982	39.98/39.25/39.52/39.94	257.0953/285.0903/303.1011	268.1034	438.1555
I	571.1811	571.1823	38.42/38.55	257.0954/285.0901/303.101	269.0885	439.1396
G	586.192	586.1929	36.67/37.60/37.86/38.10	257.0964/285.0914/303.1021	284.0992	454.1515
m2G	600.2077	600.209	39.12	257.0964/285.0913/303.1021	298.1146	468.1666
Gm	600.2077	600.209	38.67/39.12	257.0957/285.0910/303.101	298.1146	454.1483
m1G	600.2077	600.209	36.72/36.99/37.30/37.78/38.20	257.0957/285.0910/303.101	298.1146	468.1666
m7G	600.2077	600.209	36.9	257.0957/285.0910/303.101	298.1146	468.1666
